# Analysis of interactions between posaconazole/voriconazole and venetoclax

**DOI:** 10.1128/aac.01101-25

**Published:** 2026-03-31

**Authors:** Chunqing Liu, Xiaoyan Xie, Mei Sun, Pengzhou Hang, Jinxiu Lyu, Yibing Shi, Xiang Gao, Wenjun Xu, Hua Zhu

**Affiliations:** 1 School of Basic Medicine and Clinical Pharmacy, China Pharmaceutical University 56651, Nanjing, China; 2 Faculty of Pharmacy, Northern Jiangsu People's Hospital 370089, Yangzhou, China; 3 Department of Hematology, Northern Jiangsu People's Hospital 370089, Yangzhou, China; 4 College of Pharmacy, Dalian Medical University 534814 , Dalian, China; University Children's Hospital Münster, Münster, Germany

**Keywords:** myeloid neoplasms, venetoclax, posaconazole, voriconazole, blood concentration monitoring, interaction

## Abstract

Venetoclax (VEN), a selective BCL-2 inhibitor predominantly metabolized by CYP3A4, is a cornerstone therapeutic for myeloid neoplasms (MNs). Patients with myeloid malignancies are at elevated risk of invasive fungal infections (IFIs), and triazole antifungal drugs, such as posaconazole (PCZ) and voriconazole (VCZ), are commonly used for prophylaxis or treatment. These agents are potent CYP3A4 inhibitors and will exhibit significant potential for pharmacokinetic drug–drug interactions with VEN. Although studies on their interaction are limited, such combinations are frequently used in clinical practice, making further research highly significant. This study aimed to investigate the changes in blood concentration and the safety of VEN when combined with triazole antifungal drugs (PCZ and VCZ). Patients with MN treated with VEN from April 2023 to April 2025 were enrolled and allocated to the VEN monotherapy group and the VEN plus triazole antifungal drug group. We collected baseline demographic characteristics and monitored adverse events. Steady-state plasma concentrations of VEN were quantified using the liquid chromatography–mass spectrometry methodology. Statistical analyses, including comparative assessments of plasma concentrations and adverse event rates, were performed using IBM SPSS Statistics 26. A total of 54 patients were enrolled in the study. Following VEN dose reduction to 100 mg, plasma concentrations in the VEN + PCZ/VCZ group remained significantly elevated compared to the VEN group (*P* < 0.001). However, the magnitude of this elevation did not differ significantly between the VEN + PCZ group and the VEN + VCZ group (*P* = 0.176). In addition, there was no linear correlation between VEN concentration and PCZ/VCZ concentration. Safety analysis revealed no statistically significant differences between the two groups in the incidence of grade ≥3 hematological adverse events (*P* = 0.214) or severe (grade ≥3) gastrointestinal adverse events (*P* = 0.671). VEN combined with PCZ or VCZ resulted in significantly higher VEN exposure without a corresponding increase in severe hematological or gastrointestinal toxicity. This strategy effectively mitigates IFI risk without compromising the safety profile of VEN therapy.

## INTRODUCTION

Myeloid neoplasms (MNs) are a group of clonal disorders originating from hematopoietic stem or progenitor cells. This category encompasses several malignancies, including myelodysplastic syndrome (MDS), acute myeloid leukemia (AML), myeloproliferative neoplasm (MPN), and overlap syndromes, such as MDS/MPN (1). With the widespread use of targeted therapy and immunotherapy, the prognosis and survival rates of patients with hematological malignancies have significantly improved. However, due to pancytopenia after the blood test results and decreased immune function in patients with hematological malignancies, the risk of invasive fungal infections (IFIs) increases. IFIs are one of the leading causes of death in patients with hematological malignancies. Studies show that the mortality rate associated with IFIs remains as high as 50% after hematopoietic stem cell transplantation (HSCT) (2). Since IFIs lack specific clinical manifestations in the early stage and are difficult to diagnose, the guidelines recommend prophylactic use of antifungal drugs for patients at high risk of IFIs (3, 4).

**Fig 1:**
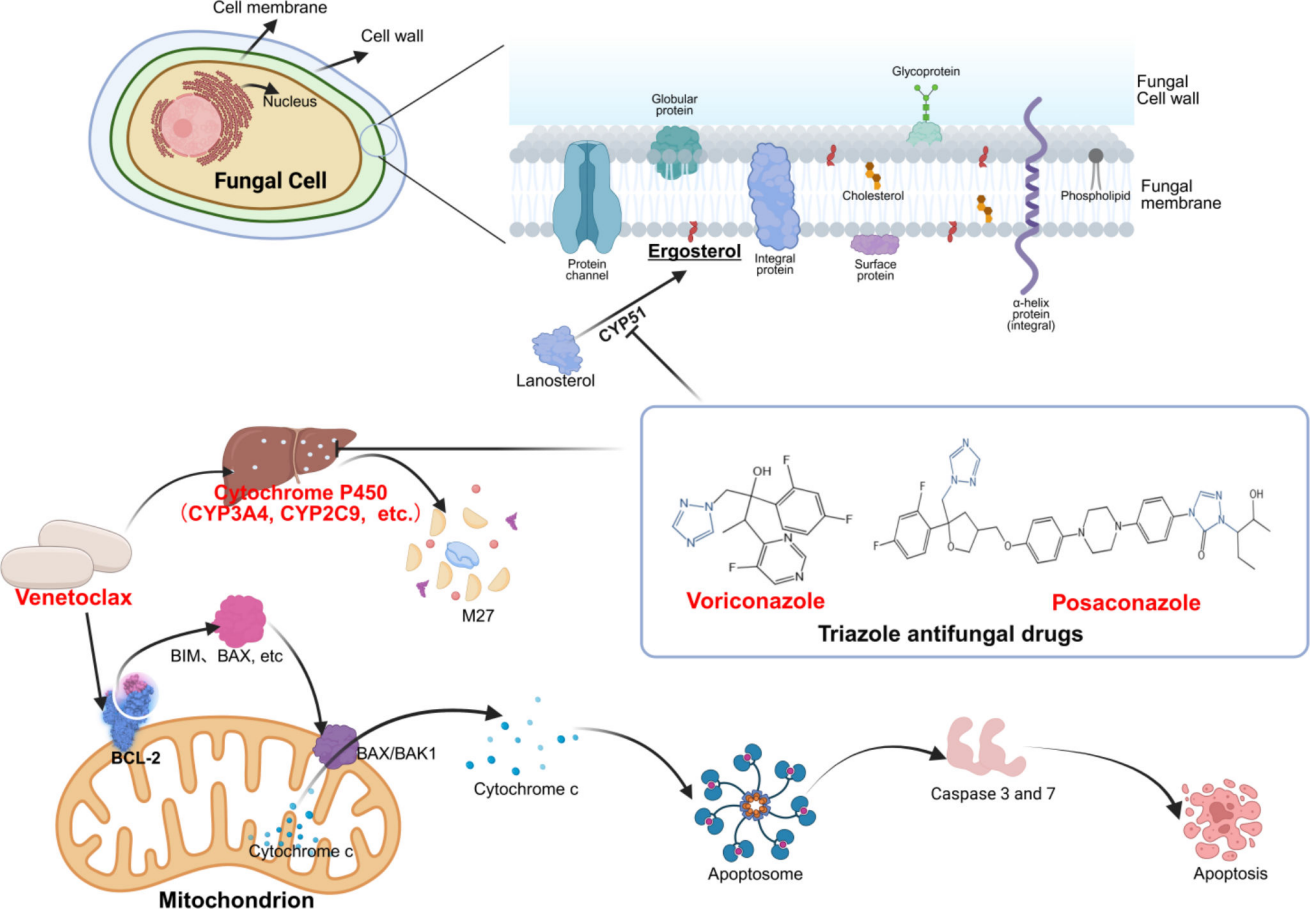
Illustration of the antifungal mechanisms of VEN and triazoles and the interaction mechanisms between them. VEN binds directly to BCL-2 protein, displacing and releasing pro-apoptotic proteins such as Bim and Bax. These released free pro-apoptotic proteins interact, initiating a cascade of apoptosis reactions that alter mitochondrial membrane permeability and release cytochrome c, further activating caspase, ultimately leading to malignant cell apoptosis. PCZ and VCZ inhibit the conversion of lanosterol to ergosterol by inhibiting the CYP51 enzyme, thereby suppressing fungal cell membrane synthesis and exerting an antifungal effect. Additionally, they inhibit the activity of CYP450 enzymes in human liver cells, thus interacting with VEN metabolized by the CYP450 enzymes, slowing down the metabolism of VEN.

IFIs refer to a condition where fungi invade the human body, grow, and multiply in tissues, organs, or blood, leading to inflammatory responses and tissue damage. Common pathogens causing IFIs in hematological patients are *Candida* and *Aspergillus* (5). Triazole antifungal drugs (hereinafter referred to as triazoles), such as voriconazole (VCZ), posaconazole (PCZ), and itraconazole, are currently recommended as first-line treatments for IFIs (4). Triazoles primarily work by binding to cytochrome P450 (CYP450) enzymes, blocking the C14α-demethylation reaction of lanosterol catalyzed by CYP450, thereby reducing ergosterol synthesis and inhibiting the structure and function of fungal cell membranes, leading to antifungal effects (6). Due to their ability to inhibit multiple CYP enzyme activities and affect the transport of drugs by P-glycoprotein in the intestine, they are prone to causing drug–drug interactions. Venetoclax (VEN) is the world’s first high-affinity oral BCL-2 inhibitor. It promotes the release of pro-apoptotic proteins (such as Bim, Bak, and Bax) by binding to BCL-2, thereby inducing apoptosis (1, 7). VEN is metabolized by CYP 3A. When co-administered with potent CYP 3A inhibitors, the exposure can increase by 1.44–6.90 times (8). Therefore, when used in conjunction with strong CYP3A4 inhibitors such as PCZ and VCZ, the risk of adverse reactions (ADRs) may increase due to elevated blood drug concentrations. The mechanism of interaction between VEN and PCZ or VCZ is shown in Fig. 1.

Currently, most studies on VEN focus on its efficacy in treating MN. There are few studies on the interactions between VEN and triazoles. Since PCZ and VCZ are frequently used antifungal agents for hematological cancer patients, understanding their interactions with commonly used drugs in the hematological system is particularly important. This study involves 54 patients with MN who were treated with VEN alone or in combination with PCZ or VCZ by analyzing their blood drug concentrations and ADR to provide a reference for selecting personalized treatment regimens for future MN patients.

## MATERIALS AND METHODS

### Research objectives

A single-center, retrospective study was performed including patients with MN admitted to the Northern Jiangsu People’s Hospital, China, from April 2023 to April 2025. Inclusion criteria included (i) age ≥18 years at the time of enrollment; (ii) morphologically, immunophenotypically, cytogenetically, and/or molecularly confirmed diagnosis of MN, including AML, MDS, MPNs, and MDS/MPN; (iii) having received at least one cycle of VEN in combination with a hypomethylating agent (HMA) as the backbone antitumor therapy; (iv) concomitant use of VCZ or PCZ for either prophylaxis or treatment of IFIs; and (v) therapeutic drug monitoring (TDM) of VEN, VCZ, and PCZ performed at steady state according to institutional protocol. Exclusion criteria included (i) TDM samples drawn at non-steady-state time points or assays failing internal quality-control criteria; (ii) co-existing gastrointestinal disorders that could impair drug absorption or tolerability; and (iii) incomplete clinical or laboratory data precluding endpoint evaluation. According to whether the included patients received concomitant triazole antifungal therapy or not, we classified them into the VEN monotherapy group (VEN group) and the VEN plus triazole group (VEN + PCZ/VCZ group).

### Drug selection and dosage regimen

Enrolled patients received oral venetoclax tablets (Venclexta; AbbVie Inc., North Chicago, IL, USA) at 400 mg once daily for ≥14 days. Treatment-naïve subjects underwent a 3-day dose ramp-up: 100 mg on day 1, 200 mg on day 2, and 400 mg daily thereafter. When VEN was combined with triazoles, the daily dose was reduced to 100 mg. Posaconazole oral suspension (Boruisa; Hunan Kelun Pharmaceutical Co., Ltd., Yueyang, China) was administered as prophylaxis at 15 mL per day (5 mL three times daily) or as treatment for IFIs at 20 mL per day (10 mL twice daily). Voriconazole tablets (Beijing SL Pharm Co., Ltd., Beijing, China) were given at an oral loading dose of 400 mg every 12 h on day 1, followed by 200 mg every 12 h in patients weighing ≥40 kg; patients weighing <40 kg received a loading dose of 200 mg every 12 h on day 1, then 100 mg every 12 h thereafter.

### Data collection

Through the hospital information management system, steady-state plasma concentrations of VEN and triazoles (PCZ and VCZ) were collected in all eligible patients. Demographic and baseline data—including sex, age, height, body weight, body mass index, underlying diseases, blood cancer types, ADR, and relevant laboratory parameters—were also retrieved: hemoglobin, platelet count, white blood cell count, neutrophil count, aspartate transaminase (AST), alanine transaminase (ALT), total bilirubin, direct bilirubin, albumin, and serum creatinine concentrations. ADRs were graded according to the Common Terminology Criteria for Adverse Events version 6.0 (9). Hematological AEs of grade ≥3 were defined as serious hematological toxicities. Serious gastrointestinal toxicities were defined as the requirement for parenteral nutrition, >7 episodes of diarrhea per day, or refractory constipation.

### Sample collection and bioanalysis

When the patient had taken a continuous dosage of VEN for at least 7 days, VCZ for at least 5 days, and PCZ for at least 7 days, we collected 5 mL of venous blood from the antecubital vein 0.5 h before morning medication administration. The blood samples were placed in tubes with EDTA. Concentration determination was performed using liquid chromatography–mass spectrometry. The mass spectrometer used was the Triple Quad4500MD model and produced by AB SCIEX Corporation in the United States, and the chromatograph was the LC20-AD model produced by Shimadzu Corporation in Japan.

### Statistical analysis

Statistical evaluation of the data was performed using SPSS 26.0 (IBM Corp.). GraphPad Prism 10.0 (Dotmatics) was used to create figures. In the present study, independent sample *t*-tests (for data that obey normal distribution and homogeneity of variance) or Mann–Whitney *U* test (for data that obey non-normal distribution) was used. Results were presented as mean ± standard deviation or median (range). Count data were presented as the number of cases (%). Independent sample *t*-tests and Mann–Whitney *U* test were used to compare the differences of the same continuous variable between different groups. Comparisons of categorical variables between groups were made using the *χ*^2^ test. The correlation analysis was performed using Pearson’s correlation *t*-test. *P* < 0.05 was considered to indicate a statistically significant difference.

## RESULTS

### Population characterization

A total of 54 patients were enrolled: 27 received VEN and 27 received VEN + PCZ/VCZ. Baseline characteristics were comparable between the two groups, except for ALT (*P* = 0.002), AST (*P* = 0.005), and the prevalence of diabetes mellitus (*P* = 0.011). The characteristics of the patients are detailed in Table 1.

**TABLE 1:** Patient characteristics and study indicators^*a*^

Characteristics	VEN group (*n* = 27)	VEN + PCZ/VCZ (*n* = 27)	*P* value
Sex, *n* (%)			0.280
Male	12 (44.44)	16 (59.26)	
Female	15 (55.56)	11 (40.74)	
Age (years)	67 (58–69)	71 (58–75)	0.092
HT (cm)	162.60 ± 8.61	166.97 ± 7.08	0.432
BW (kg)	63.04 ± 10.02	65.25 ± 9.00	0.256
BMI (kg/m^2^)	23.79 ± 3.12	23.38 ± 2.61	0.960
Hb (g/L)	90.96 ± 26.52	94.44 ± 26.15	0.701
WBC (×10^9^ L^−1^)	4.44 (1.97–5.36)	3.31 (1.45–5.67)	0.653
PLT (×10^9^ L^−1^)	87 (40–156)	51 (30–137)	0.406
ANC (×10^9^ L^−1^)	1.13 (0.36–3.59)	2.08 (0.34–3.06)	0.703
Renal function			
Scr (µmol/L)	70.30 ± 17.4	81.42 ± 22.87	0.133
Liver function			
ALT (U/L)	23.70 (17.70–34.30)	12.30 (8.50–19.90)	0.002
AST (U/L)	21.10 (17.80–32.30)	16.00 (11.30–20.90)	0.005
TBIL (g/L)	9.40 (7.50–12.10)	10.50 (7.80–13.70)	0.324
DBIL (g/L)	4.00 (3.20–5.00)	4.90 (3.20–6.90)	0.206
ALB (g/L)	38.00 ± 6.01	38.91 ± 5.21	0.339
Tumor types, *n* (%)			0.386
AML	25 (92.59)	23 (85.19)	
MDS	2 (7.41)	4 (14.81)	
Underlying conditions, *n* (%)			
Hypertension	9 (33.33)	9 (33.33)	1.000
Diabetes mellitus	1 (3.70)	8 (29.63)	0.011

^*a*^ ALB, albumin; ALT, alanine transaminase; AML, acute myelocytic leukemia; ANC, neutrophil count; AST, aspartate transaminase; BMI, body mass index; BW, body weight; DBIL, direct bilirubin; Hb, hemoglobin; HT, height; MDS, myelodysplastic syndrome; PLT, platelet count; Scr, serum creatinine concentration; TBIL, total bilirubin; WBC, white blood cell count.

### Effects of triazoles on VEN blood concentration

**Fig 2:**
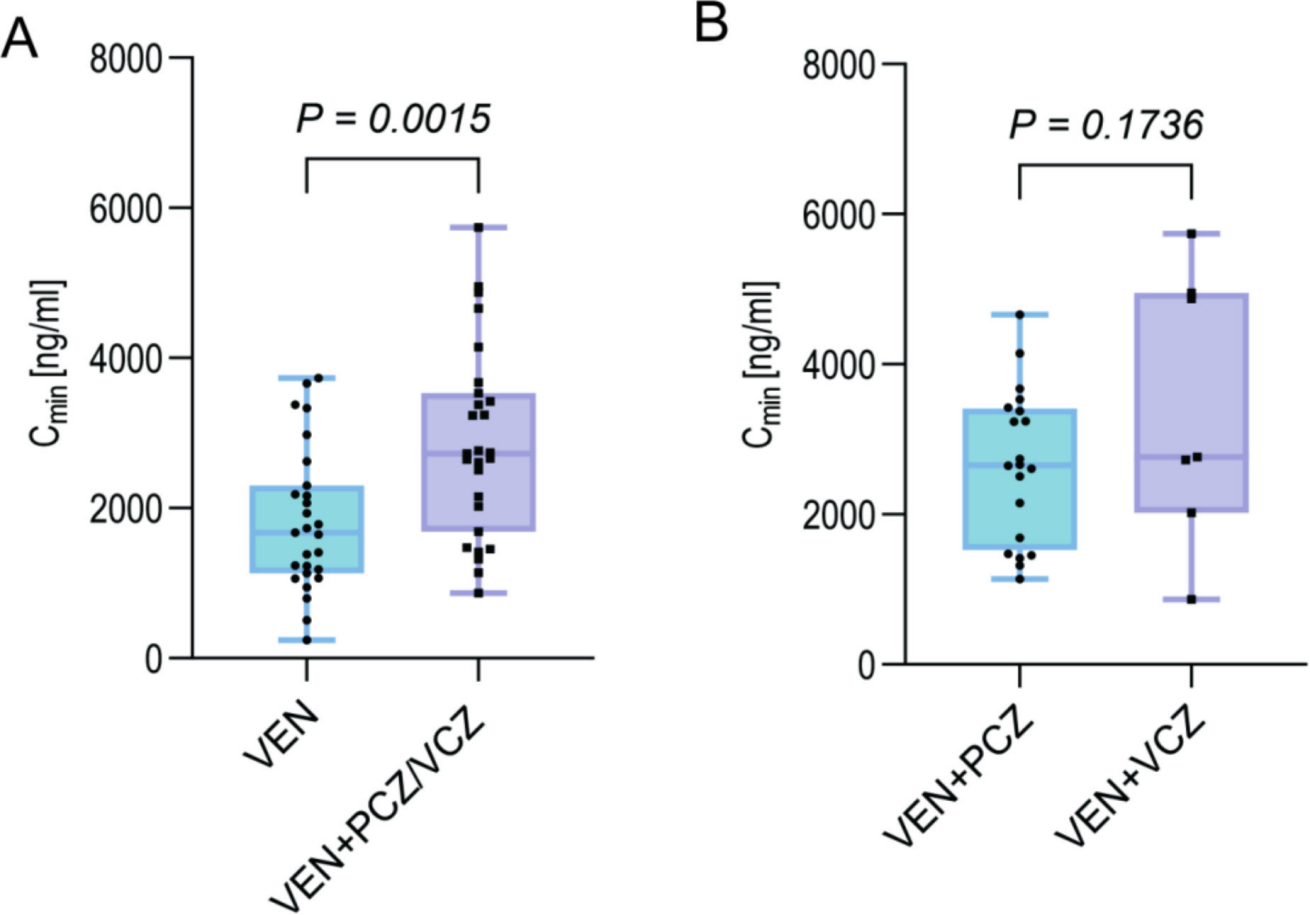
Box plot of the effect of PCZ/VCZ combination on VEN blood concentration. (**A**) Comparison of VEN blood concentration between the combination group and the monotherapy group. (**B**) Comparison of the effects of PCZ and VCZ on VEN blood concentration.

After excluding samples that had not reached steady state or were affected by analytical error, 54 VEN concentration measurements were retained. Independent-sample *t*-test showed that, even after the VEN dose was reduced to 100 mg daily, trough concentrations in the PCZ/VCZ group remained significantly higher than in the VEN group (2,853.52 ± 1,267.34 ng/mL vs 1,828.73 ± 950.00 ng/mL; *P* = 0.001; Fig. 2A). However, whether PCZ and VCZ exert differential effects on VEN exposure has not been established. To address this, we compared the VEN + PCZ group and the VEN + VCZ group. Mann–Whitney *U* test revealed no statistically significant difference in VEN concentrations between the two triazoles (median IQR: 1,883.41 ng/mL vs 2,928.12 ng/mL; *P* = 0.176; Fig. 2B).

**Fig 3:**
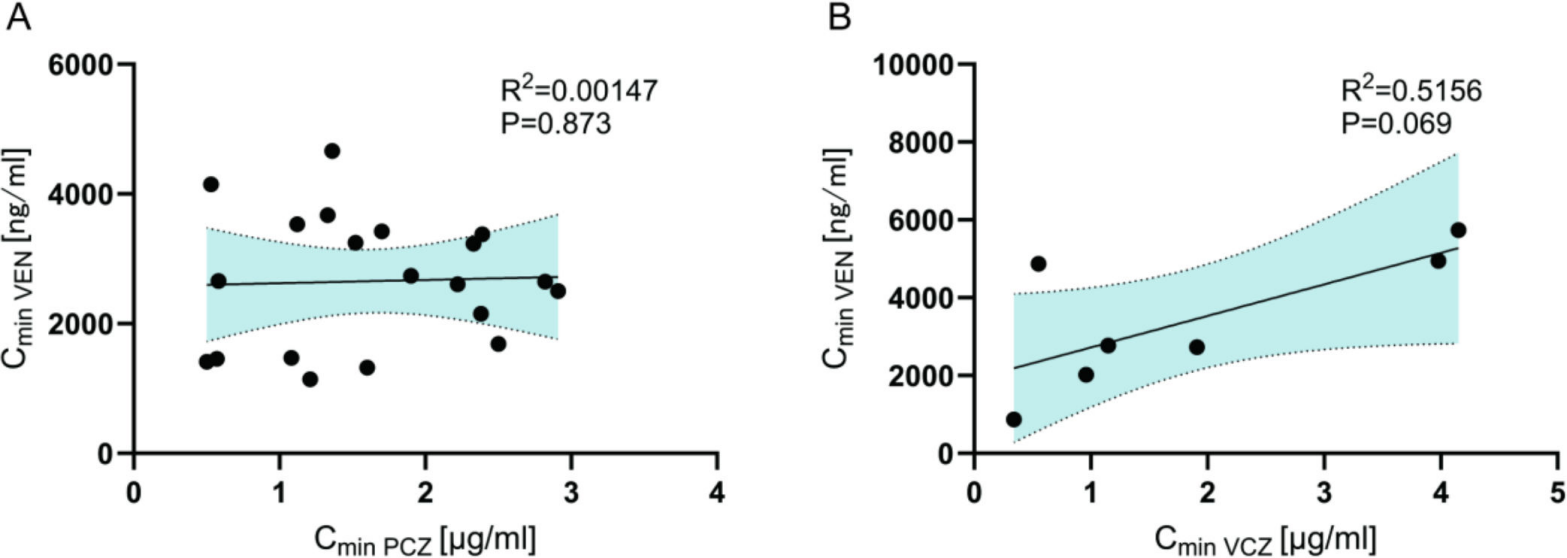
Correlation between PCZ/VCZ blood concentration and VEN blood concentration. (**A**) Correlation between PCZ and VEN blood concentration. (**B**) Correlation between VCZ and VEN blood concentration.

To verify whether VEN exposure increases linearly with triazoles concentrations, we performed correlation analyses in the VEN + PCZ group and in the VEN + VCZ group. As shown in Fig. 3, VEN trough levels did not rise indefinitely with increasing PCZ (Fig. 3A) or VCZ (Fig. 3B) concentrations.

### Safety analysis

In the VEN group, 25 patients experienced hematological ADRs, including 18 patients with grade 3 or higher hematological ADRs, and 10 patients experienced gastrointestinal ADRs (including nausea, diarrhea, constipation, and abdominal pain), with 6 patients experiencing severe gastrointestinal ADRs. In the VEN + PCZ/VCZ group, 25 patients experienced hematological ADRs, including 22 patients with grade 3 or higher hematological ADRs, and 8 patients experienced gastrointestinal ADRs, with 4 patients experiencing severe gastrointestinal ADRs. *χ*^2^ test was conducted on the patients who experienced grade 3 or higher hematological ADRs and severe gastrointestinal ADRs in both groups, revealing no significant differences between the two groups for grade 3 or higher hematological adverse reactions (*P* = 0.214, Table 2) and severe gastrointestinal ADRs (*P* = 0.671, Table 2).

**TABLE 2:** Hematological and gastrointestinal ADRs in the VEN group and the PCZ/VCZ group

	VEN (*n* = 27)		VEN + PCZ/VCZ (*n* = 27)		*χ* ^2^	*P* values
	All grades *n* (%)	≥Grade 3 *n* (%)	All grades *n* (%)	≥Grade 3 *n* (%)		
Hematological ADRs	25 (92.59)	18 (66.67)	25 (92.59)	22 (81.48)	1.543	0.214
Leukopenia	23 (85.19)	15 (55.56)	23 (85.19)	18 (66.67)	–^*a*^	–
Neutropenia	21 (77.78)	15 (55.56)	23 (85.19)	17 (62.96)	–	–
Anemia	21 (77.78)	11 (40.74)	20 (74.07)	11 (40.74)	–	–
Thrombocytopenia	16 (59.26%)	13 (48.15)	22 (81.48)	18 (66.67)	–	–
Gastrointestinal ADRs	10 (37.04%)	6 (22.22)	8 (29.63)	4 (14.81)	0.180	0.671

^*a*^ –, not applicable.

## DISCUSSION

Numerous studies have shown that VEN, when used in combination with azacitidine or other hypomethylating agents, is well tolerated in patients with MN or HSCT, significantly improving median overall survival, complete remission rates, and composite complete remission rates (1, 10–13). However, there are relatively few studies on the efficacy and safety of VEN in combination with triazoles. Guo et al. and Wang et al. separately reported retrospective studies conducted in northern China, showing that VEN concentrations were significantly higher when it was co-administered with PCZ or VCZ than in patients receiving VEN alone (14, 15). Through a non-randomized, open-label, phase 1b study, DiNardo et al. showed that PCZ was estimated to increase VEN maximum observed plasma concentration by 7.1 times, which also supported the use of antifungal prophylaxis with PCZ in patients with the disease who are receiving VEN after reducing the VEN dose by at least 75% (10). Agarwal et al. and Hall et al. noted that ketoconazole can increase the *C*_max_ and the AUC_∞_ of VEN by 2.3 and 6.4 times, respectively (16, 17). Similarly, using a physiologically based pharmacokinetic (PBPK) model, Agarwal et al. and Dong et al. predicted that VCZ could increase the exposure of VEN by 4.5–9.6 times (16, 18). Furthermore, Agarwal et al. and Bhatnagar et al. used the PBPK model to find that when co-administered with 300 mg once-daily extended-release PCZ tablets, the exposure of VEN was consistent with actual data. Additionally, when the dose of PCZ was increased to 500 mg, the exposure of VEN increased about 12% but remained within the safe range. Therefore, it is recommended that the dose of VEN be adjusted to 70 mg when co-administered with PCZ (16, 19). This study evaluated the safety of VEN monotherapy versus its concomitant use with PCZ or VCZ in patients from southern China. Trough VEN concentrations were significantly higher in the combination group than in the monotherapy group, indicating that co-administration with triazoles—even after dose reduction to 100 mg daily—may still result in excessive VEN exposure. No significant difference in VEN levels was observed between the PCZ and VCZ subgroups, which may be related to the limited sample size. Additionally, we studied the correlation between VEN concentration and triazole concentration. The analysis revealed a non-linear relationship, suggesting that the enzymatic inhibition mediated by triazoles may become saturated at higher concentrations.

In general, the higher the blood drug concentration, the greater the risk of ADRs, but most literature indicates that an increase in VEN blood concentration does not lead to an increased risk of ADRs. Hall et al. noted that although the dose of VCZ needs to be adjusted when used in combination with VEN, it did not result in worse hematological outcomes (17). This suggests that reasonable dose adjustments can balance the benefits of antifungal prophylaxis and cancer treatment. Özkocaman et al. found that adding PCZ to the regimen of VEN and azacitidine significantly reduced fungal infections without increasing the risk of ADRs (20). Rausch et al. pointed out that combining VEN with triazoles prolongs platelet recovery time but has no significant impact on neutrophil recovery (21). This study analyzed the incidence of ADRs between the monotherapy group and the combination therapy group, finding no significant difference in the risk of grade 3 or higher hematological ADRs and severe gastrointestinal reactions between the two groups. Therefore, the combination of these two drugs reduces the probability of fungal infection and treatment costs for patients without increasing the likelihood of hematological ADRs caused by VEN, ensuring the safety, efficacy, and cost-effectiveness of clinical use.

In clinical practice, it is crucial to manage the rational use of VEN in combination with other drugs, particularly CYP3A inhibitors. Mukherjee et al. found through PBPK and population pharmacokinetic modeling that existing dose adjustment guidelines for VEN remain valid even when CYP3A inhibitors are used concurrently (22). The findings of this study further support this view, but the study has several limitations: first, the sample size was small, which may introduce errors in conclusions regarding the effects of different triazoles; second, being a single-center study, the conclusions may be somewhat biased. Future research should consider conducting multi-center studies, increasing the sample size, further exploring whether there is an enzyme saturation phenomenon of triazoles inhibiting VEN and model-based individualized medication regimens, and enhancing awareness of therapeutic drug monitoring to optimize the efficacy and safety of combination therapy.

### Conclusion

In the treatment of MN, VEN combined with HMA is a common regimen. However, due to the immunocompromised state of these patients, the risk of fungal infections is higher, so it is necessary to use triazoles concurrently for prevention or treatment. Studies have shown that when triazoles are used in combination with VEN, they not only do not reduce the efficacy of MN treatment but also effectively lower the risk of fungal infections and reduce chemotherapy costs. Additionally, this combination does not significantly increase the risk of ADRs. Therefore, in future MN treatment strategies, clinicians are advised to consider incorporating VEN and triazoles into the treatment plan after a comprehensive assessment of the patient’s condition, to achieve better therapeutic outcomes and higher cost-effectiveness, bringing more clinical benefits to patients.

## Data Availability

The data sets generated and/or analyzed during the current study are available from the corresponding author upon reasonable request.
